# Comparative assessment of malaria rapid diagnostic tests (RDT) in Ibadan, Nigeria

**Published:** 2017-10-01

**Authors:** Rose I. Ilesanmi, Oluwasogo A. Olalubi, Oluwasegun T. Adetunde, Ayodele O. Ilesanmi, Hyacinth Effedua, Abimbola O. Amoo

**Affiliations:** 1 Department of Medical Microbiology, Olabisi Onabanjo University, Ago-Iwoye, Nigeria; 2 Department of Public Health, Kwara State University, Malete, Nigeria; 3 Department of Geography and Environmental Management, University of Ilorin, Ilorin, Nigeria; 4 Department of Medical Laboratory Science, Kwara State University, Malete, Nigeria

## Abstract

**Background:**

Deployment of sound diagnostic tests remains a crucial component of malaria management, prevention and control in Africa. We undertook a comparative assessment of sensitivity, specificity and efficiency of three popular brands of rapid diagnostic tests (RDT) available in Nigerian market alongside with traditional microscopy.

**Materials and methods:**

525 samples of patients that presented with acute uncomplicated malaria through clinical diagnosis were evaluated with the various tests. Total WBC count and haematocrit were also measured.

**Results:**

Of the 525 samples, 300 (57.1%) were found positive by Giemsa microscopy. SD Bioline had a positivity rate of 49.5% (260/525), while the positivity rate for Acon was significantly lower (38.1%; 200/525) and Paracheck (28.6%; 150/525). The sensitivity, specificity and efficiency of the three RDTs were: SD Bioline (86.3%, 99.6%, 92%); Paracheck (50%, 97.7%, 70.4%) and Acon (66.7%, 100%, 80.9%), respectively. Pre-teens aged 6-12 yrs had the highest mean malaria parasite densities with 6,631.26 at p< 0.01. The dominant malaria species was *Plasmodium falciparum* with 280 (93.3%) cases. Co-infections of *P. falciparum/vivax* (15; 5.0%) and *P. falciparum/malariae* (5; 1.7%) were detected and confirmed with microscopy. Haematocrit values correlated inversely with parasite density (r = -0.744; *p*< 0.01).

**Conclusions:**

Microscopy still remains the reference standard for malaria diagnosis in limited resource settings in endemic areas. In furtherance to this, there is need for consistent monitoring of RDT product quality as part of the distribution process to end-users across Nigeria.

## 1 Introduction

Malaria remains one of mankind’s most dreaded diseases, with 212 million cases and more than 400 thousand deaths reported globally in 2015 [1]. Although recent estimates suggest that global mortality decreased by an impressive 47% between 2000 and 2013, and by 54% in Africa, it still remains a major public health burden in many countries. Africa accounted for 90% of malaria cases [1] and about 40% of deaths that occurred in just two countries: Nigeria and DR Congo [2].

Nigeria, with a population of 134 million, records more than 100 million cases and a mortality of 300 thousand annually [3]. Efforts to control malaria with insecticide treated bednets seem not to yield the desired result, possibly due to wrong use or abuse of the nets, low rate of ownership and coverage, poor or low user compliance, low literacy levels and commitment to adhere to training received from past malaria control programmes. Although malaria is curable, its endemicity and re-occurrences, coupled with its high mortality in children [2] and pregnant women, pose a major challenge in Africa and especially in Nigeria.

One of the challenges in malaria management, particularly for children, is inaccurate diagnosis [4]. Clinical diagnosis of malaria in a region with many tropical infectious diseases has limited reliability since these have many signs and symptoms in common. Clinical diagnosis of malaria without laboratory support may lead to misdiagnosis and wrong treatment [5]. Drug resistance compounds this situation and one of the control strategies to combat malaria is to improve diagnosis and treatment [6]. Although examination of a thick blood smear after Giemsa staining remains the preferred method for malaria diagnosis it is labour-intensive and time consuming. Moreover, as reliable as this technique is, few cases may still be missed. Nonetheless, microscopy offers a lot of advantages over other diagnostic techniques. Microscopic examination of blood films for malaria is only feasible in standard laboratory settings, which are often absent in remote rural areas. Consequently, the use of rapid diagnostics (RDT) for malaria diagnosis has become widespread. For instance [7] advocated the use of RDTs at community pharmacies in Nigeria. Continuous influx of imported RDT kits into the Nigerian market with little or no regulation is a cause for concern and has the subtle tendency to compromise malaria management and control. The assumption that all RDT kits work similarly and exhibit high efficiency is misplaced and has a tendency to worsen efforts aimed at malaria control in Nigeria.

The aim of this study was to undertake a comparative assessment of the efficiency of three popular brands of RDTs found in the Nigerian market alongside standard microscopy. The goal was to determine the efficiency and relative strength of each product in detecting malaria so as to enable end users to make informed choices. This in turn should aid or enhance better quality assurance and control of laboratory diagnostics for malaria and ultimately promoting better health care delivery.

## 2 Materials and methods

### 2.1 Study area

The study was carried out in Glory Diagnostica Laboratories Ojoo, Ibadan, the capital of Oyo State in southwestern Nigeria, dominated by the Yoruba ethnic group. The study was conducted between June and December 2012. During this period rainfall is relatively heavy in the area. Prevailing ecological and socio-cultural practices and the poor socioeconomic status of residents make malaria to be endemic throughout the year.

### 2.2 Study design and population

The study adopted a non-randomised descriptive and cross-sectional study. In all, a total of 525 participants were recruited and diagnosed for acute uncomplicated malaria during medical consultation. Patient samples were obtained for RDT and Giemsa-stained microscopic slides from Mount Zion, Fajimi Memorial, Mosolape, Olufunmilayo and Olushola Hospitals, all situated in Ojoo area of Ibadan, Nigeria.

Sample size was determined using the method of [8]. Briefly, using a prevalence of 50.0%, precision 0.05(5%) and statistical power of 80%, the minimum sample size was 223 subjects. This was increased due to technical capacity of the research team in sample collection and analysis. Individuals with fever and without focus (foci) of infection [9] were included in the study; those without other visible signs of fever were also included. Individuals with either confirmed severe, chronic complicated malaria or with sepsis as concomitant infection were excluded.

### 2.3 Sample collection and analysis

Before sampling, temperature of each patient was measured with the aid of a mercury-type clinical thermometer. Demographic data, i.e. the age and sex of patients were recorded. Five (5) ml of venous blood from each patient was collected into a clean plastic EDTA container for haematocrit value determination and total white blood cell (WBC) count. Thin and thick blood films were Giemsa-stained for malaria microscopy. At the beginning of each test procedure, individual patient blood samples were thoroughly mixed and tested for malaria antigen reactivity separately with Acon, SD Bioline or Paracheck kits. Known positive and negative controls for malaria parasite were run along with patients’ test samples. Haematocrit or packed red cell volume (PCV) were performed by filling a plain capillary tube to ¾ levels with blood from an EDTA container by capillary flow. One end of the capillary tube was sealed with plasticine and placed in a haematocrit centrifuge and spun for 5 minutes at 5,000 revolutions per minute. The spun capillary tube was read with a haematocrit reader. Normal PCV values ranged from 40-55% and 36-45% for males and females, respectively.

Thick film smears on three spots were made from fresh blood samples. The films were properly dried (without prestaining fixing) and then stained with 10% Giemsa solution and subsequently washed (after 10 min) using buffered distilled water (pH 7.2). A drop of immersion oil was applied on the dried stained slide and examined microscopically for malaria parasites using 100x oil immersion objective lens. Asexual parasite observation was regarded as a positive smear; smears were considered negative after examination of 100 high power fields did not reveal parasites. An experienced medical laboratory scientist stained the slides with Giemsa using standard methods [10] and parasites counted against 200 white blood cells. Similarly, thin blood films were prepared for *Plasmodium* species identification, quantifying parasitaemia, and recognition of other parasite forms like schizonts and gametocytes.

Two laboratory scientists independently examined the slides; a third reading of discordant results by another senior scientist was considered final. Consistent quality assurance activities were put in place to ensure that microscopy was conducted by competent and motivated medical staff supported by effective training and supervision and an adequate supply of reagents and equipment. Blood smears from patients with suspected malaria were routinely screened, blindly rechecked and validated by senior microscopists. Second and third slide external blind readers were used to ensure that discordant results were further read and final results produced. Compared to baseline assessment the first-round external quality assessment found a remarkable improvement in the quality of microscopic malaria diagnosis, from 64 to 87% agreement between slide readings.

Of each blood sample 0.02 ml was added to 0.38 ml of Turk’s solution and gently mixed. After 5 min the mixture was charged into a Neubauer counting chamber and allowed to settle for 2 min before counting was done under x10 objective of the microscope.

### 2.4 Parasite density estimation

A portion of the thick film where white cells were evenly distributed and parasites well stained was selected for examination. 200 White blood cells (WBCs) were systematically counted. Asexual forms of the parasites in the field covered were estimated concurrently. The number of parasites per μl of blood was calculated using the WHO formula [11]:


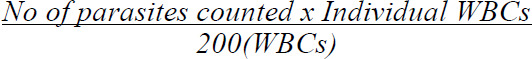


*200(WBCs)*

A slide was declared negative when there was no parasite detected/counted within 200 WBCs. Two laboratory scientists independently examined the slides; the third reading of discordant results by another scientist was taken as final.

### 2.5 RDT kits and quality control

**SD Bioline®:** The test device was removed from the foil pouch and brought to room temperature. 20μl of whole blood was added to the sample well(s) of the test device. 3 drops of assay diluent was then added. The timer was set and a pink colour developing within 10 minutes indicated a positive result. The presence of two or three colour bands (‘1’, ‘2’ and c within the result window, no matter which appears first, indicates a positive result for Pf (*P. falciparum*) or Pv (*P. vivax*) and positive control, respectively.

**Paracheck (Pf):** Paracheck (Pf) kit components were brought to room temperature before testing. 5μl of anticoagulated blood was delivered into sample well A. Six (6) drops of the clearing buffer was delivered into well B. The mixtures are allowed to react. The timer was set and a pink colour within 15 minutes denoted a positive result.

**Acon:** The test strip was removed from the foil pouch and used immediately. 10μl of whole blood was transferred into a specimen tube and 3 drops of buffer was added into the same specimen tube. The test strip was immersed into the contents of the test tube, taking caution not to exceed the maximum line on the test strip. The timer was set and a pink colour within 10 min indicated a positive result. Two distinct coloured lines appeared. One in the control (c) and the other in the test region. This indicated a positive result for Paracheck/Acon. A negative was indicated by no coloured line appearing in the test region but only in the control region (c).

The RDTs used in this evaluation were made available through the Ministry of Health and Social Welfare and included SD Bioline®, Paracheck (Pf) and Acon kits obtained through the existing national procurement and distribution system. Log books and unique ID stickers were provided to record RDT results and for specimen tracking. The test is only limited to the detection of antigens of *P. falciparum.* The entire test kits were within the shelf life (Manufacturing date December 2012 and expiry date May 2015) and were stored in the laboratory store which was air-conditioned, albeit not on a 24 hour basis. Hence, the temperature was variable. Recommended storage temperature by the manufacturer is between 1°C and 40°C. The microscopy and RDT were done by different laboratory personnel. Each facility received training on how to appropriately collect, label and store specimens. Health workers at all participating facilities were trained to perform RDTs.

### 2.6 Data analysis

Pearson’s product quotient correlation analysis was used to determine the relationship between haematocrit values and malaria parasite density in the sampled population. This determined if haematocrit levels influenced occurrence of malaria in the region. Standard error of means was generally used in a descriptive role to access distribution of data such as parasite densities by age groups and *Plasmodium* species. Analysis of variance (ANOVA) was used to determine the influence of specific age groups on malaria parasite densities. This helped to determine the age group with the highest prevalence of malaria. The receiver operating characteristics (ROC) curve was used to evaluate which of the diagnostic methods yielded the best malaria diagnosis. This was plotted and interpreted using the area under the curve.

### 2.7 Ethical approval

Oral informed consent was obtained from the selected patients. Ethical approval was obtained from Olabisi Onabanjo University Teaching Hospital, Ogun State, Nigeria and Kwara State University Research Committee and Ethical Review Board. Individuals were assured of voluntary participation, confidentiality of their test results and opportunity to withdraw at any time without prejudice, in line with the Helsinki Declaration [12].

## 3 Results

The observed difference in relative positivity between males and females (56.3% and 58.2%, respectively) was not significant (Table 1), indicating equal susceptibility to malaria for both sexes in the study area. This result corroborates what was observed in past research [13]. The observed slight difference of (+1.9%) in females could be due to release of sex-specific hormones in the female body [14]. Females compared to males have been known to have lower immunity to malaria and higher mortality from malaria especially when pregnant [15,16].

**Table 1:** Malaria positivity and mean parasite density according to sex.

		Number	%	%	Mean parasitaemia
Sex	Total	Positive	Positive	Relative positivity	±SEM
Males	286	161	53.7	56.3	3297±539
Females	239	139	46.3	58.2	2866±475
Total	525	300	57.1		

There was a statistically significant difference across the age groups with regards to parasite density (Table 2). There was an observed weak positive relationship between malaria parasite density and age. Partial Eta square was 0.086 and F=6.459 (P< 0.01).

**Table 2:** Relationship between age and parasite density in sample population.

Source	Type III sum of squares	Df	Mean square	F	Sig.	Partial eta squared
Corrected model	9.780E8	4	2.445E8	6.459	P<0.01	0.086
Intercept	2.644E9	1	2.644E9	69.835	P<0.01	0.202
Age group	9.780E8	4	2.445E8	6.459	P<0.01	0.086
Error	1.045E10	276	3.785E7			
Total	1.461E10	281				
Corrected total	1.143E10	280				

a. R^2^ = 0.086 (Adjusted R^2^ = 0.072)

Table 3 shows parasite densities by age group. The highest parasite densities were found between age 6-12 years followed by under five children (P<0.01). The observed low parasite density in patients over 50 years is due to acquired immunity developed by virtue of repeated exposure to malaria parasites over the years.

**Table 3:** Parasite density of sample population by age group.

			99% Confidence interval	
Age group	Mean	Std. error	Lower bound	Upper bound
< 5yrs	4691.720	679.438	2929.420	6454.019
6-12yrs	6631.257	1039.975	3933.812	9328.702
13-19yrs	4086.345	1142.505	1122.963	7049.727
20-50yrs	1632.901	583.977	118.206	3147.596
>50yrs	1202.958	1255.889	-2054.516	4460.433

*Plasmodium falciparum* was the most prevalent species (93.3%; Table 4). Co-infection for *P. falciparum / P. vivax* as well as *P. falciparum / P. malariae* were observed in 5% and 1.7% of the samples, respectively. The mean parasite densities across all identified species were highest in the co-infection samples. Observed densities for *P. falciparum* was 2,245 parasites per μl of blood while co-infections *P. falciparum / P. vivax* and *P. falciparum / P. malariae* were 15,917 and 13,520 parasites per μl of blood respectively.

**Table 4:** Malaria positivity by *Plasmodium* species.

Species	No. positive (%)	Mean parasite density (±SEM)
*P. falciparum*	280 (93.3)	2245±328
*P. falciparum/P. vivax*	15 (5.0)	15917±813
*P. falciparum/P. malariae*	5 (1.7)	13520±781
Total	300 (100.0)	

The frequency at which fever occurred in malaria positive patients is shown in Table 5. It was observed that 242 (89%) of malaria positive patients had fever while 58 (22.9%) did not show signs of fever. On the other hand, 195 (77.1%) tested negative for malaria and did not have fever. However, 30 (11.0%) patients who tested negative to malaria had fever. This observation could be attributed to fever as symptom of other diseases like pneumonia, urinary tract infection, typhoid, etc. The relationship between having fever and testing positive for malaria was significant (P<0.05).

**Table 5:** Frequency of subjects with fever among malaria positive patients.

Malaria	Yes (%)	No (%)	Total
Positive	242 (89.0)	58 (22.9)	300
Negative	30 (11.0)	195 (77.1)	225
Total	272 (100.0)	254 (100.0)	525

*X*^2^=233.6, P<0.05

Table 6 shows the relationship between haematocrit values and malaria parasite density in samples. The Pearson’s 2 -tailed correlation analysis was carried out at P<0.01. It was observed that there was a strong negative relationship (r = - 0.744) between haematocrit values and malaria parasite density in the collected samples. As such, the higher the number of healthy red blood cells the lower the malaria parasite density and vice versa.

**Table 6:** Comparison of haematocrit values in malaria and non-malaria subjects.

		Haematocrit value	Parasite density
Haematocrit value	Pearson correlation	1	-0.744^**^
	Sign. (2-tailed)		0.000
	Sum of squares and cross-products	32248.296	-1.117E7
	Covariance	61.660	-40039.877
	N	525	281
Parasite density	Pearson correlation	-0.744^**^	1
	Sign. (2-tailed)	0.000	
	Sum of squares and cross-products	-1.117E7	1.142E10
	Covariance	-40039.877	4.093E7
	N	281	281

** P<0.01

Giemsa thick smears had the highest malaria positivity of 300 (57.1%) and negativity of 225 (42.9%) of total samples examined (Table 7). This was followed by SD Bioline RDT kit with a malaria positivity of 260 (49.5%) and negativity of 265 (50.5%) of total samples examined. The least observed malaria positivity was Paracheck RDT kit with 150 (28.6%) and negativity of 375 (71.4%) of samples examined. The Giemsa reference standard was seen to be a better diagnostic tool for malaria diagnosis than the RDT.

**Table 7:** Relative malaria positivity by RDT kit used.

		RDT			
	Giemsa thick smear N (%)	Acon N (%)	SD Bioline N (%)	Paracheck N (%)
Positive	300 (57.1)	200 (38.1)	260 (49.5)	150 (28.6)
Negative	225 (42.9)	325 (61.9)	265 (50.5)	375 (71.4)
Total	525 (100)	525 (100)	525 (100)	525 (100)

**Figure 1:**
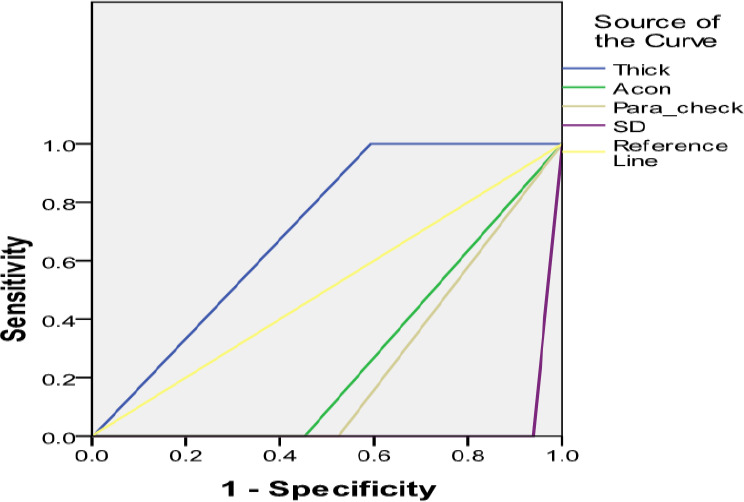
Receiver Operating Characteristic curve (ROC) for the 3 RDTs and Giemsa thick smear (‘Thick’) diagnostic methods. Sensitivity (y-axis) is the true positive rate; the x-axis shows the false positive rate (‘1-Specificity).

Table 8 shows SD Bioline to have the highest efficiency and sensitivity of the RDT kits; it had a sensitivity, specificity and efficiency of 86.3%, 99.6% and 92.0%, respectively. This was followed by Acon RDT kit with sensitivity and efficiency of 66.7% and 80.9% respectively while having the highest specificity of 100%. Paracheck RDT kit performed the least with sensitivity, specificity and efficiency of 50.0%, 97.7% and 70.4%, respectively. Figure 1 shows the prediction accuracy of both microscopy technique as well as the use of RDT kits in testing for malaria. From the receiver operating characteristic (ROC) curve, thick film microscopy predicted with 70.1% area under the curve for detection of malaria parasites. This was followed by the RDT kits with Acon 27.3%, Paracheck 23.7% and SD Bioline 3.1% at p<0.05 respectively.

**Table 8:** Sensitivity, Specificity and Efficiency of the tested RDT kits (in %).

RDT	Acon	SD Bioline	Paracheck
Sensitivity	66.7	86.3	50.0
Specificity	100.0	99.6	97.7
Efficiency	80.9	92.0	70.4

## 4 Discussion

Early diagnosis and prompt treatment of malaria, especially in rural areas or at primary health care (PHC) level is vital to combating the disease in all endemic regions [1,17]. In addition, Nigeria had set for itself the year 2015 to achieve one of the millennium development goals (MDGs), which was to combat the trio of deadly infectious diseases – malaria, tuberculosis and the human immunodeficiency-virus (HIV) infection. One of the ways to control any infectious disease is to cure the infection in affected patients, in order to limit its spread [17]. To achieve this effectively, patients are required to be diagnosed promptly and correctly, combining both clinical and laboratory tools. In recent times, efforts at achieving this goal has led to the increased use of rapid diagnostic test (RDT) kits for malaria diagnosis, particularly in the field and rural communities where laboratory facilities are absent [7].

This study compared the efficiency of three different but commonly used rapid diagnostic test (RDT) kits, namely Acon, Paracheck and SD Bioline along with Giemsa microscopy as the reference standard. When malaria positivity for 525 specimens were analysed using different RDTs alongside the Giemsa microscopy method, the latter was able to detect highest number of positive malaria cases. The best of the RDT kit (SD Bioline) had a malaria positivity of 260 (49.5%) of total samples diagnosed. Its positivity relative to the Giemsa method was 86.7%. Results obtained from this study confirmed that microscopy remains the reference standard and a better diagnostic tool for malaria diagnosis in the laboratory than the RDTs.

The specificity (99.6%) observed with SD Bioline was almost similar to the observation made in the study of [18] in whose report the SD Bioline specificity was 100%. Also, the sensitivity and specificity obtained in this study with Paracheck were dissimilar to the observation of [19] who found Paracheck to be 92.6% sensitive and 98.6% specific. However, [18] and [19] worked with a single type kit only. Observed disparity in the result obtained with Paracheck in this study and that of [19] is still not clear, especially since all necessary precautionary measures were observed in terms of maintenance of optimum temperature and storage conditions so as to protect the quality of the test kits.

SD Bioline is seen as the best RDT kit of the three kits analysed in this study, and is able to detect low level parasitaemia. This makes it more sensitive than the others. Besides, it has a sensitivity and specificity of 86.3% and 99.6%, respectively. Moreover, it was generally observed that the higher the parasite density, the heavier the colour intensity. In other words, the degree of parasitaemia may be estimated by the extent of colour produced on the test strip, implying that in field work, the intensity of strip colour during/after testing with this kit may be used to give a rough assessment of parasite density in the patient.

In considering malaria infection according to patients’ age, the highest level of parasitaemia was observed in the age group (6-12) years. This could be as a result of the naïve nature of the immune status coupled with low blood volume as well as other comorbid factors such as malnutrition, within that age bracket, making children more susceptible to malaria infection. It is pertinent to note that [18] recorded similar results, with their highest level of parasitaemia recorded in the 1-5 yrs age group, using only the SD Bioline kit.

Furthermore, *P. falciparum* was found to be the most predominant species among the infecting parasite species, constituting 93.3% of total malaria cases identified in this study. This is in consonance with the submission of [20] that in many tropical countries *P. falciparum* is the predominant species. *P. falciparum* infections are known to be highly pathogenic and since it is the most prevalent in this study, it may account for why malaria disease takes a great toll on the Nigerian population. Co-infection with other malaria parasite strains accounted for the remaining (6.7%). There was an observed 9.3% recorded for other malaria strains in past work by [21]. The observed inverse relationship between haematocrit values and malaria parasite density (r = - 0.744, *p* < 0.01) was in line with [22] who had similar results of low haematocrit values with malaria positive subjects. A high parasite density implies that a greater number of red blood cells are parasitized peripherally with a consequence of higher red blood cells destruction leading to haemolytic anaemia and severe fever. Outside peripheral parasite count, presence of intraleucocytic malaria pigment found in neutrophils also indicate disease severity [23]. This connotes that haematocrit value determination is very paramount and should be conducted along with malaria parasite testing, especially among children presenting with worse case prognosis [24].

With a temperature of >37.5°C taken as the benchmark for fever, two hundred and forty-two (242) subjects, representing 89.0% had fever symptoms out of 300 found positive for malaria parasite (p<0.05). This suggests that among people with pyrexia in the tropics, malaria parasite screening should be one of the key tests to be conducted while not excluding other probable causes. A previous study recorded 894 (55%) positive cases out of 1640 subjects having fever symptoms [25].

It is pertinent to mention one important diagnostic advantage in microscopy which RDTs lack - the ability of the analyst to observe the morphological features of the parasite under the microscope. This makes it possible for the microscopist to identify different parasite forms and stages commonly seen under the microscope, a feature which has a lot of implications for the diagnosis of critical parasite forms like schizonts or gametocytes. The inability of RDTs to detect such parasite forms may scales down the gravity of detection of infection especially during severity, thereby contributing directly or indirectly to morbidity and mortality. Several pitfalls have been listed in using RDTs for the diagnosis of malaria [6]. This included possibility of cross reactions of the antigens in the immunochromatographic strip with rheumatoid factor, autoantibodies and certain other non -malarial infections. Furthermore, false positive results for *Plasmodium* species that are absent in blood when *P. falciparum* is in high concentration. In addition, the continued presence of *p*HRP-2 antigens in blood several weeks after treatment, even when parasites have been cleared from the blood and inability to fairly assess the degree of parasitaemia, among others, should be watched out for.

However, the establishment and maintenance of a reliable and efficient diagnostic facility for malaria at the primary health care level is dependent on many (or a combination of) factors, which can seriously degrade the utility and relevance of any malaria laboratory. These can be broadly summarised as follows: (a) Deficiencies in personnel adhering to adequate work time management, (b) substandard or inappropriate equipment: incorrect or poorly coordinated specifications, particularly of microscopes; irregular maintenance and replacement of worn-out parts; weak quality control of stains and reagents; inappropriate chain supply schedules which either cause delays in the supply of material or accumulate stocks which might exceed the shelf-life of the material, (c) organisational deficiencies: the maintenance of an efficient and relevant organisational structure calls for frequent review and analysis of the current aims and objectives of the malaria diagnostic facility as well as the necessary changes in staffing and methods to meet these requirements with available resources [26]. From time to time this may involve radically changing deficient personnel and other unused resources and overcoming the inertia to change at local levels.

## 5 Conclusions

Giemsa staining technique has been reconfirmed in this study to be the best reference routine method for malaria diagnosis. Assessment of results from this study has shown SD Bioline as the best RDT kit among the three kits assessed, being the kit with highest sensitivity, specificity and efficiency. It may thus be used where facilities for microscopy does not exist.

Although *Plasmodium falciparum* was the more prevalent malaria species among the subjects, higher parasite density was more common in co-infections. Demonstration of higher parasite density has shown that children within the age 6-12 years are at high risk of malaria infection. Results from this study indicate that the degree of malaria parasitaemia correlates inversely with the patient’s haematocrit level. Low haematocrit values found among malaria-infected subjects demonstrated that fever and anaemia are one of the major symptoms of malaria infection in Ibadan.

Although SD Bioline has shown a proven efficiency in the diagnosis of malaria in this study, it can only be recommended as a preliminary adjunct to Giemsa staining technique in malaria diagnosis. Laboratorians in developing countries should continue to employ the Giemsa quantitative analysis for routine purposes so as to achieve effective diagnosis, disease control and efficacious treatment of malaria. It is imperative that malaria microscopy, being a reliable, affordable and accessible technique be assessed regularly against emerging technologies for malaria diagnosis.
